# Hospitalary outbreak of *Burkholderia cepacia* bacteremia associated with a decrease of chlorination of water system

**DOI:** 10.1186/1753-6561-5-S6-O76

**Published:** 2011-06-29

**Authors:** A Galeana, C Scarpita, N Ramos, G Leiva, H Albornoz, MM Godino

**Affiliations:** 1 Hospital Maciel, Uraguay; 2 FNR, Montevideo, Uruguay

## Introduction

*Burkhordelia Cepacia* (BC) complex is a genomic-related group of gram negative bacilli. Haemato oncological (HO), and dialysis patients (pts) are especially vulnerable to microorganisms that contaminate the hospital environment, especially water.

## Objectives

Report a clonal prolonged outbreak of bacteraemia by BC in a tertiary hospital in Uruguay.

## Methods

Description and analysis of the outbreak, genetic identification of the organism by molecular biology and communicating the interventions.

## Results

Between 27/3/2008 and 3/2/2008, 4 pts in the HO unit developed BC bacteremia.

In March 2008, 6 ptes developed chills during haemodialysis (HD), blood cultures didn’t grow BC. At 15/4/2008 another patient in HO unit developed bacteraemia by BC.

In the HO unit the molecular biology confirmed a clonal outbreak. The crops of antiseptic solutions, soap, tap water, and bottled water showed no growth of BC.

Measurements of the levels of chlorine in tap water was less than or equal to 0.25 ppm. Subsequently the chlorination deficit was confirmed at the water system throughout the hospital.

The final molecular biology analysis confirmed the clonality of the bacteremia in the 5 HO pts, one peak of dialysis (11 and 19 March), a HD machine and the tip of a dialysis catheter. A shock chlorination of the water system of hospital was performed.

## Conclusion

*- A prolonged*, *and spreaded clonal outbreak of BC was manifested in the pts who are especially vulnerable.*

*- The organism was identified in ptes and devices*, *and in the water system associated with decreased chlorination*, *.*

- The chlorination of the water system was an effective measure to control the outbreak.

## Disclosure of interest

None declared.

